# The revised Canadian Bleeding (CAN-BLEED) score for risk stratification of bleeding trauma patients: a mixed retrospective—prospective cohort study

**DOI:** 10.1186/s13049-025-01336-z

**Published:** 2025-02-20

**Authors:** Alexandre Tran, Tyler Lamb, Shannon M. Fernando, Manya Charette, Marie-Joe Nemnom, Maher Matar, Jacinthe Lampron, Christian Vaillancourt

**Affiliations:** 1 Division of General Surgery, Department of Surgery, University of Ottawa, Ottawa, Canada; 2 Acute Care Research Program, Ottawa Hospital Research Institute, Ottawa, Canada; 3 Division of Critical Care, University of Ottawa, Ottawa, Canada; 4 Department of Critical Care, Lakeridge Health Corporation, Oshawa, Canada; 5 Department of Emergency Medicine, University of Ottawa, Ottawa, Canada; 6 The Ottawa Hospital, Civic Campus, 1053 Carling Avenue, Ottawa, ON K1Y4E9 Canada; 7 Department of Critical Care, The Ottawa Hospital, Ottawa, Canada

**Keywords:** Trauma, Hemorrhage, Clinical decision aids, Prognostication

## Abstract

**Background:**

Traumatic hemorrhage is a significant cause of morbidity and mortality. There is considerable interest in risk stratification tools to aid with early activation of intervention pathways for bleeding patients. In this study, we refine the Canadian Bleeding (CAN-BLEED) score for the prediction of major interventions in bleeding trauma patients.

**Methods:**

We conducted a mixed retrospective-prospective cohort study. We included a retrospective cohort from the CAN-BLEED derivation study, from September 2014 to September 2017. We also conducted a prospective cohort from May 2019 to August 2021 and included both datasets for refinement of the CAN-BLEED score. The primary outcome was major intervention, defined by a composite of massive transfusion, embolization, or surgery for hemostasis. Predictors were pre-specified based on previous validation work. We used a stepdown procedure and regression coefficients to create a clinical risk stratification score. We used bootstrap internal validation to assess optimism-corrected performance.

**Results:**

We included 1368 patients in the overall cohort. Incidence of penetrating injury was 23% and median injury severity score was 17. The overall incidence of the need for major intervention was 17%. The revised score included 8 variables: systolic blood pressure, heart rate, lactate, penetrating mechanism, pelvic instability, Focused Abdominal Sonography for Trauma positive for free fluid, computed tomography positive for free fluid, or contrast extravasation. The C-statistic for the simplified score is 0.89. A score cut-off of less than 2 points yielded a 97% (94–98%) sensitivity in ruling out the need for major intervention.

**Conclusion:**

The revised CAN-BLEED score offers a clinically intuitive and internally validated tool with excellent performance in identifying patients requiring major intervention for traumatic bleeding. Further efforts are required to evaluate its performance with an external validation.

**Supplementary Information:**

The online version contains supplementary material available at 10.1186/s13049-025-01336-z.

## Background

Traumatic hemorrhage is a significant cause of morbidity and mortality among patients worldwide, with estimates suggesting that it accounts for up to 40% of all trauma deaths [1]. Uncontrolled bleeding is the most common cause of preventable mortality following military and civilian trauma [2]. Management of hemorrhage requires prompt recognition, damage control resuscitation, and early hemostasis [3]. However, severe blood loss often occurs insidiously, leading to clinical uncertainty, delayed intervention, and worse patient outcomes [4, 5].

For these reasons, there has been significant research interest in clinical decision aids to prognosticate outcomes among bleeding patients [6–8], especially to guide activation of massive transfusion protocols [9, 10]. However, there are concerns related to competing risk and survivorship bias for scores designed to predict massive transfusion alone [11]. The CAN-BLEED score was derived and internally validated for the prediction of any major intervention including massive transfusion protocol delivery, hemostatic surgery, or embolization in trauma patients [12]. Due to limitations in available sample size during the initial derivation study, the prespecified model was not able to incorporate all potential variables of interest.

The objective of this study is to refine the CAN-BLEED score for the prediction of major interventions in bleeding trauma patients, combining a previous retrospective cohort with a prospective cohort to attain sufficient sample size to evaluate all variables of interest.

## Methods

### Design and setting

We conducted a mixed retrospective-prospective observational cohort study at The Ottawa Hospital Civic Campus. The Ottawa Hospital is the designated Level 1 trauma center, university-affiliated hospital, and regional referral center for the Champlain Local Health Integration Network in Ontario, Canada. We included a retrospective observational cohort from the initial CAN-BLEED derivation study, from September 2014 to September 2017 [12]. In addition, we conducted a prospective observational cohort from May 2019 to August 2021, and included both datasets for refinement of the CAN-BLEED prediction model.

### Retrospective Cohort

We included a previously published retrospective observational cohort from the CAN-BLEED derivation study, conducted from September 2014 to September 2017 [12]. In that study, we included 890 adult patients arriving alive to the Ottawa Hospital with blunt or penetrating trauma to the thorax, abdomen, or pelvis requiring trauma team activation. Patients were eligible if received from scene or from a transferring hospital within 3 h from time of injury. Patients were excluded if they had an out-of-hospital cardiac arrest or suffering from non-hemorrhagic mechanisms of injury (e.g., burn injury, drowning, or electrocution). We completed preliminary derivation and internal validation of the CAN-BLEED score to identify patients with clinically significant bleeding—defined as the need for massive transfusion, angioembolization or surgery for hemostasis. However, we did not have a sufficient number of events to incorporate all potential variables of interest such as mechanism and heart rate. Importantly, models constructed with an event per variable ratio of 10 or less are at higher risk of potential overfitting, which results in models that may deliver a strong performance during initial derivation but typically perform poorly at external validation [13]. As such, we additionally conducted a prospective observational cohort with the intention to combine datasets for refinement of the CAN-BLEED score.

### Prospective cohort

In the prospective cohort, we utilized the same eligibility criteria from the prior study. We enrolled 478 adult patients arriving alive to the Ottawa Hospital with blunt or penetrating trauma to the thorax, abdomen, or pelvis requiring trauma team activation and concerns for potential traumatic bleeding between May 2019 and August 2021.

Following confirmation of patient eligibility, our study team reviewed the electronic medical record to collect standardized information on key time points, clinical characteristics, and outcomes. This was completed by a trained abstractor and an audit of the first 100 cases followed by 10% of all cases thereafter was conducted for quality assurance. Formal inter-rater reliability was not calculated. We additionally cross-referenced our abstracted data with that from our institutional trauma registry to ensure consistency with regards to case identification and calculation of the injury severity score. This is maintained by a dedicated data analyst, with a standardized input form and routine quality assurance audits consistent with all trauma centres across the province of Ontario.

The primary outcome of interest was need for major intervention, which was defined as any of the following: massive transfusion, angioembolization or surgery for hemostasis (collectively intended to represent clinically significant bleeding). To classify surgical outcomes, we reviewed all operative reports for eligible patients and included those requiring thoracotomy, laparotomy, pelvic packing or fixation, and vascular surgery for the specific intention of hemostasis. Patients receiving an exploratory operation without a therapeutic hemostatic procedure within the operative report were classified as non-events. In the initial derivation study, we utilized the classical definition for massive transfusion (≥ 10 PRBCs in 24 h). However, in this refinement study, massive transfusion protocol delivery was defined as activation of the massive transfusion protocol and receipt of at least 3 units packed red blood cells (PRBC) within the first hour (critical administration threshold) for both the retrospective and prospective datasets. This definition was utilized to minimize the survivorship or competing risk bias associated with traditional massive transfusion definitions of at least 6 or 10 units over 24 h [11]. Patients receiving angiography without embolization were also classified as not meeting the primary outcome.

### Data analysis and model creation

The data analysis protocol was developed in accordance with guidelines for clinical prediction modeling [14–17]. The retrospective and prospective cohorts were merged into a single dataset and reviewed for data integrity. Similar to the methodology employed for the CAN-BLEED derivation study, we noted that the variables for Lactate (56% missing), FAST (30% missing) and CT (11% missing) were not missing at random [12]. All other variables had less than 5% missing values. Importantly, missing values for Lactate, FAST and CT were more likely to be available in patients that were clinically unwell (with abnormal findings) and more likely to be missing in those that were clinically well (with unremarkable findings). As such, when these values were missing, we considered them to be unremarkable (Lactate < 5, negative FAST, no free fluid or extravasation on CT). In accordance with best practice methodological guidance [18], the appropriateness of this assumption was investigated by comparison with imputed values generated under a multivariable imputation model. All remaining continuous variables were modelled using restricted cubic splines. We used a multiple imputation technique to impute missing values on predictor variables using the aregImpute function in the Hmisc library [19]. This procedure simultaneously imputes missing variables while determining optimal transformations among all imputation variables. The imputation model consisted of the full list of predictor variables, outcomes, and available ancillary variables (such as age, sex, and time to hospital).

We prioritized full pre-specification of the predictors, use of flexible functions for continuous predictors, and multiple imputation. Model pre-specification, based on clinical knowledge and evidence from the literature, minimizes the risk of over-fitting in small datasets and improves performance at external validation [20]. The variables reviewed for determination of model pre-specification were based on clinical importance as determined by a prior systematic review [6, 7], as well as national [8] and international surveys [21] conducted by our group. We used multiple imputation for missing values using the aregImpute function in the Hmisc library [19], which models continuous variables using restricted cubic splines with three default knots, while categorical variables are expanded as dummy variables representing all categories within a categorical variable. We used Variance Inflation Factors to examine multi-collinearity.

An initial, fully pre-specified logistic regression model was fitted on the first imputed dataset using spline functions with five knots for the two continuous variables. Next, a plot of partial associations referred to as an ANOVA plot, corrected for the number of degrees of freedom, was generated to visualize strong and weak partial associations. The strengths of association are used to inform a decision of how many degrees of freedom to allocate to each variable in the final model: strong associations are modeled with greater complexity than weak associations. The fully pre-specified model with nine predictors was fitted to the 10 multiple imputation datasets and the results were combined across the datasets using Rubin’s rules. Harrell's fast stepdown procedure was run on the previous full model [19]. Stepdown models improve practicality with fewer predictors for use in clinical practice. No variable was deleted. Analyses were conducted using R version 4.2 and SAS version 9.4 software.

### Score simplification

From the stepdown model, a nomogram was generated, and the number of points associated with each predictor were obtained from the estimated regression coefficients to create a simplified score. For continuous predictors, the points were determined to correspond to midpoints of clinically meaningful intervals of values. We used a receiver operative curve analysis to assess sensitivity, specificity, positive predictive value (PPV), negative predictive value (NPV), and area under the curve for the simplified score as compared to the full complex model.

### Internal validation

Harrell et al*.* presented an algorithm to assess the degree of overfitting resulting from the model building process [19]. In this algorithm, the model is estimated separately using each bootstrap sample and subsequently evaluated in the original sample to calculate optimism. Bootstrap validation maximizes sample size while minimizing potential overfitting—as opposed to split or cross validation, which is considered less statistically efficient [20]. We generated 1000 bootstrap samples and calculated optimism-corrected performance. The C-statistic for the simplified model was compared to that of the full model and the reduced model to ensure minimal change in performance.

### Human ethics and consent to participate declaration

This study was approved by the Ottawa Health Science Network Review Ethics Board. A waiver of consent from participants was obtained.

### Funding declaration

This study was funded by an operational grant from the Ottawa Hospital Academic Medical Organization Innovation Fund.

## Results

We included 1368 patients in the overall cohort—890 patients from the retrospective derivation cohort and an additional 478 patients from the prospective cohort. The population characteristics are presented in Table 1. Most patients were males (76%) with a median age of 42 years (interquartile range [IQR] 27–58). There was a 23% incidence of penetrating injury and a median injury severity score was 17 (IQR 10–26). The overall incidence of major intervention, defined as a composite of surgery for hemostasis, embolization, or massive transfusion, was 17%. The population characteristics were similar between the retrospective derivation cohort and the prospective cohort, though we note there was a higher incidence of penetrating injury (25% vs 22%), visualized bleeding (12% vs 5%), and need for major intervention (21% vs 15%) in the prospective cohort.

**Table 1:** Population characteristics

Characteristic	Initial derivation cohort (n = 890)	Prospective cohort (n = 478)	Overall (n = 1368)
Age (years), median (IQR)	43 (27 to 58)	39 (26 to 58)	42 (27 to 58)
Male, n (%)	675 (76%)	370 (77%)	1,045 (76%)
Penetrating Mechanism of Injury, n (%)	192 (22%)	120 (25%)	312 (23%)
*Baseline clinical characteristics, median (IQR)*			
Initial systolic blood pressure (mmHg)	125 (110 to 140)	126 (106 to 142)	125 (110 to 140)
Initial heart rate (beats/minute)	91 (77 to 108)	90 (77 to 107)	91 (78 to 108)
Glasgow Coma Scale Score	15 (14 to 15)	15 (12 to 15)	15 (14 to 15)
Injury Severity Score, median (IQR)	17 (10 to 25)	17 (9 to 26)	17 (10 to 26)
Pelvic Instability, n (%)	42 (5%)	27 (6%)	69 (5%)
Visualization of active bleeding, n (%)	41 (5%)	58 (12%)	99 (7%)
FAST performed, n (%)	545 (61%)	418 (87%)	963 (68%)
FAST positive	92 (10%)	83 (17%)	175 (13%)
Hemoglobin (g/L), median (IQR)	138 (125 to 150)	142 (128 to 153)	139 (126 to 151)
pH, median (IQR)	7.33 (7.26 to 7.37)	7.33 (7.28 to 7.39)	7.33 (7.26 to 7.38)
Base excess (mmol/L), median (IQR)	− 1.5 (− 5.1 to 1.1)	− 0.1 (− 3.4 to 2.2)	− 1.1 (− 4.8 to 1.5)
Lactate level (mmol/L), median (IQR)	3 (2 to 4.7)	3 (2.2 to 4)	3 (2.1 to 4.5)
CT imaging performed, n (%)	787 (88%)	436 (91%)	1223 (89%)
Free fluid on CT	219 (25%)	146 (31%)	365 (27%)
Contrast extravasation on CT	74 (8%)	76 (16%)	150 (11%)
*Interventions, n (%)*			
Any major intervention	133 (15%)	101 (21%)	234 (17%)
Surgery	102 (11%)	74 (15%)	176 (13%)
Embolization	35 (4%)	11 (2%)	46 (3%)
Massive transfusion	45 (5%)	41 (9%)	86 (6%)

IQR, interquartile range; FAST, focused abdominal sonography for trauma; CT, computed tomography

### Full Pre-specified and reduced models

The full pre-specified model is presented in the *Supplement*. The C-statistic for the full model was 0.90 and remained at 0.90 after the stepdown procedure, demonstrating excellent performance. After 1000 bootstrap samples for internal validation, the optimism-corrected C-statistic was 0.90. The reduced multivariable model, after using the stepdown procedure, included eight variables: initial systolic blood pressure, heart rate, lactate, penetrating mechanism, pelvic instability requiring fixation (as identified on clinical exam), Focused Abdominal Sonography for Trauma (FAST) positive for free fluid, and computed tomography (CT) positive for abdominopelvic free fluid or contrast extravasation. The modelled association for blood pressure and heart rate using restricted cubic splines is presented graphically in the *Supplement*.

### Simplified score

The resulting Canadian Bleeding (CAN-BLEED) score is revised and presented in Table 2. The C-statistic for the simplified score is 0.89 confirming excellent performance.

**Table 2:** CAN-BLEED score composition

Revised CAN-BLEED Score		
Variable	Value	Points
Initial systolic blood pressure at presentation	SBP < 90 90 ≤ SBP < 110	2 1
Visualized external bleeding	Yes	2
Unstable pelvis	Yes	1
Computed Tomography: Free fluid or active extravasation	Yes	2
Lactate > 5 mmol/L	Yes	1
Focused abdominal sonography for trauma positive	Yes	1
Initial heart rate at presentation	120 ≥ HR 100 ≤ HR < 120	2 1
Mechanism of injury	Penetrating	1

Initial CAN-BLEED Score		
Variable	Value	Points
Initial systolic blood pressure at presentation	SBP < 90 90 ≤ SBP < 110	2 1
Visualized external bleeding or Unstable pelvis	Yes	2
Computed Tomography: Free fluid or active extravasation	Yes	2
Lactate > 5 mmol/L	Yes	1
Focused abdominal sonography for trauma positive	Yes	1

SBP, systolic blood pressure; HR, heart rate

### Measures of performance

The calibration plot (*Supplement)* showed excellent agreement between observed and predicted probabilities across the entire spectrum of risk. The classification performance for the revised CAN-BLEED score is presented in Table 3*.* A score cut-off of less than 2 points yielded a 97% (94% to 98%) sensitivity in ruling out the need for major intervention.

**Table 3:** Classification table

Score	Sensitivity			Specificity		
	%	95% CI		%	95% CI	
0	100.0	98.4	100.0	**–**	–	–
1	100.0	98.4	100.0	35.9	33.1	38.8
2	97.0	93.9	98.8	58.4	55.5	61.3
3	87.6	82.7	91.5	75.0	72.4	77.5
4	70.1	63.8	75.9	86.9	84.8	88.8
5	53.9	47.2	60.4	93.4	91.8	94.8
6	36.8	30.6	43.3	97.2	96.0	98.1
7	18.4	13.6	23.9	98.9	98.1	99.4
8	4.7	2.4	8.3	99.7	99.2	100.0
9	1.7	0.5	4.3	100.0	99.7	100.0
10	0.4	0.0	2.4	100.0	99.7	100.0
11	–	–	–	100.0	99.7	100.0
12	–	–	–	100.0	99.7	100.0

CI, confidence interval

## Discussion

The revised CAN-BLEED score includes the same variables as the initial model (systolic blood pressure, lactate, FAST, CT imaging, and visualized bleeding or pelvic instability), but additionally incorporates mechanism of injury and heart rate. These variables were chosen a priori based on clinical importance as determined by a systematic review [6], as well as national [8] and international surveys of trauma care providers [21]. The score shares many of the same clinical elements as the previously developed assessment of blood consumption (ABC) [9] and Trauma Associated Severe Hemorrhage (TASH) [10] scores for prediction of massive transfusion, but offers the additional advantage of being derived and internally validated specifically for a pragmatic composite outcome representative of clinically significant bleeding. Importantly, the sole utilization of massive transfusion as an outcome of interest is prone to competing risk and survivorship bias [11, 22–24]. A recent study of pre-hospital trauma patients demonstrated that a novel algorithm, termed the Bleeding Risk Index, using continuously recorded noninvasive vital signs may offer improved predictive performance over traditional bleeding risk scores [25]. Similar to our study, the authors utilized the critical administration threshold (3 PRBCs in 1 h) to minimize the impact of survivorship bias. However, the impact of competing risk bias and performance at external validation remains unknown. Further studies comparing the predictive performance of the refined CAN-BLEED score to existing bleeding scores is required.

In an international survey of trauma care providers from Canada, United States, Germany, Australia, and New Zealand, clinicians noted challenges in executing the risk stratification, identification of need, and delivery of meaningful interventions for bleeding trauma patients [21]. Respondents linked these challenges to systems factors such as variability in the process or efficiency of pathway activations, as well as cognitive factors such as the inherent limitations in clinical gestalt for risk stratification. Clinicians have stated a willingness to adopt a well-validated and sensitive risk stratification tool for bleeding patients but to date have expressed variable confidence in the existing decision support frameworks [21].

The revised CAN-BLEED score offers a pragmatic risk stratification tool based on variables easily available to the treating provider early in the initial assessment. The score is intentionally designed to mimic the clinician’s intuitive decision-making process, as a priori models based on clinical expertise offer improved acceptance by healthcare providers [26] and superior performance at external validation [17, 20, 27]. During the early phases of initial resuscitation, high-risk patients can be identified based on hemodynamics, clinical exam and FAST alone. If such variables are within normal limits and the clinician has a low pre-test probability of clinically important hemorrhagic injury, then further investigation is typically avoided. This decision-making mirrors the real-world assumptions utilized during derivation and internal validation of our model—whereby cases without CT imaging (30%) were specifically re-coded as unremarkable. If there is a high pre-test probability of occult injury following initial assessment, clinicians often pursue additional investigation including CT imaging—a diagnostic modality increasingly advocated for by the American College of Surgeons Committee on Trauma Guidelines on Imaging and incorporated by trauma centres into the initial evaluation of major trauma patients [28].

An objective and evidence-based approach to risk stratification has meaningful clinical, resource, and academic applications. This score was derived and validated at a Canadian tertiary care centre and reflects the practice patterns of experienced, high volume trauma care providers. While its intention is not to supplant clinical expert decision-making, its pragmatic derivation and validation based clinical and research expertise may serve to provide a reliable, evidence-based framework to homogenize decision-making for less experienced providers. In addition, it may aid clinical decision-making with regards to inter-hospital transfer assessments and activation of key intervention pathways—an important area of need identified by trauma care providers [21, 25]. In addition, it may provide a more consistent and objective assessment of risk to better define the population of “at-risk bleeding trauma patients” for enrolment into clinical trials—an endeavor that has previously been limited by imprecise event estimates resulting in inefficient sample size estimates and eventual clinical trial futility [29].

### Strengths and limitations

Strengths of this study include an exhaustive evaluation of clinically important variables based on previous systematic review [6] and survey studies [8, 21], as well as adherence to best practice guidelines for derivation and validation of prediction models [14–17, 20] in order to maximize performance at external validation. In addition, we incorporated two separate datasets, including a two-year prospective observational cohort, optimizing sample size for refinement and evaluation of important variables. This study also has limitations. While the optimism-corrected performance at internal validation is impressive, this remains a single centre study at a Canadian tertiary care trauma centre. Internal validation with bootstrapping does not necessarily imply strong performance at external validation as it does not account for information or selection bias that may be not evident across different datasets [30]. In addition, bootstrap validation may deliver overly optimistic performances in small datasets due to persistent overfitting. In addition, due to meaningful missingness of data, we employed and verified assumptions of normality in such circumstances to maximize real-world pragmatism. However, the appropriateness of such assumptions can only be confirmed at external validation. We were also unable to account for time-dependent trends across the entire study period such as changes in clinical practice or composition and experience of trauma care providers. Further efforts are required to externally validate score performance at other centres in order to compare it to existing risk stratification tools and assess its potential to support clinical decision-making.

## Conclusion

We conducted a mixed retrospective-prospective observational cohort study in order to refine a pragmatic bleeding risk stratification tool for bleeding trauma patients. The revised CAN-BLEED score offers a clinically intuitive and internally validated tool with excellent performance in identifying patients requiring major intervention for traumatic bleeding. Further efforts are required to evaluate its performance by external validation.

## Supplementary Information

Supplementary file 1

## Data Availability

Partial or complete datasets and data dictionary are available upon reasonable request to Dr. Alexandre Tran (aletran@toh.ca).

## References

[1] Kauvar DS, Lefering R, Wade CE. Impact of hemorrhage on trauma outcome: an overview of epidemiology, clinical presentations, and therapeutic considerations. J Trauma. 2006;60(6 Suppl):S3-11. 16763478 10.1097/01.ta.0000199961.02677.19

[2] Shackelford SA, Colton K, Stansbury LG, Galvagno SM Jr, Anazodo AN, DuBose JJ, et al. Early identification of uncontrolled hemorrhage after trauma: current status and future direction. J Trauma Acute Care Surg. 2014;77(3 Suppl 2):S222–7. 24770559 10.1097/TA.0000000000000198

[3] Cannon JW, Khan MA, Raja AS, Cohen MJ, Como JJ, Cotton BA, et al. Damage control resuscitation in patients with severe traumatic hemorrhage: a practice management guideline from the Eastern Association for the Surgery of Trauma. J Trauma Acute Care Surg. 2017;82(3):605–17. 28225743 10.1097/TA.0000000000001333

[4] Tien HC, Spencer F, Tremblay LN, Rizoli SB, Brenneman FD. Preventable deaths from hemorrhage at a level I Canadian trauma center. J Trauma. 2007;62(1):142–6. 17215745 10.1097/01.ta.0000251558.38388.47

[5] Lamb T, Tran A, Lampron J, Shorr R, Taljaard M, Vaillancourt C. The impact of time to hemostatic intervention and delayed care for patients with traumatic hemorrhage: a systematic review. J Trauma Acute Care Surg. 2023;95(2):267–75. 36973874 10.1097/TA.0000000000003976

[6] Tran A, Matar M, Lampron J, Steyerberg E, Taljaard M, Vaillancourt C. Early identification of patients requiring massive transfusion, embolization or hemostatic surgery for traumatic hemorrhage: a systematic review and meta-analysis. J Trauma Acute Care Surg. 2018;84(3):505–16. 29251714 10.1097/TA.0000000000001760

[7] Tran A, Matar M, Steyerberg EW, Lampron J, Taljaard M, Vaillancourt C. Early identification of patients requiring massive transfusion, embolization, or hemostatic surgery for traumatic hemorrhage: a systematic review protocol. Syst Rev. 2017;6(1):80. 28407781 10.1186/s13643-017-0480-0 PMC5390372

[8] Tran A, Matar M, Lampron J, Steyerberg E, Vaillancourt C, Taljaard M. Outcome variation among Canadian trauma centres: toward a clinical prediction rule for standardizing approaches to clinical assessment of hemorrhage. Can J Surg. 2017;60(5):E3. 28930038 10.1503/cjs.1760051 PMC5608566

[9] Nunez TC, Voskresensky IV, Dossett LA, Shinall R, Dutton WD, Cotton BA. Early prediction of massive transfusion in trauma: simple as ABC (assessment of blood consumption)? J Trauma. 2009;66(2):346–52. 19204506 10.1097/TA.0b013e3181961c35

[10] Yucel N, Lefering R, Maegele M, Vorweg M, Tjardes T, Ruchholtz S, et al. Trauma Associated Severe Hemorrhage (TASH)-Score: probability of mass transfusion as surrogate for life threatening hemorrhage after multiple trauma. J Trauma. 2006;60(6):1228–36. 16766965 10.1097/01.ta.0000220386.84012.bf

[11] Tran A, Nemnom MJ, Lampron J, Matar M, Vaillancourt C, Taljaard M. Accuracy of massive transfusion as a surrogate for significant traumatic bleeding in health administrative datasets. Injury. 2019;50(2):318–23. 30448330 10.1016/j.injury.2018.11.014

[12] Tran A, Taljaard M, Abdulaziz KE, Matar M, Lampron J, Steyerberg EW, et al. Early identification of the need for major intervention in patients with traumatic hemorrhage: development and internal validation of a simple bleeding score. Can J Surg. 2020;63(5):E422–30. 33009903 10.1503/cjs.010619 PMC7608708

[13] Peduzzi P, Concato J, Kemper E, Holford TR, Feinstein AR. A simulation study of the number of events per variable in logistic regression analysis. J Clin Epidemiol. 1996;49(12):1373–9. 8970487 10.1016/s0895-4356(96)00236-3

[14] Hemingway H, Croft P, Perel P, Hayden JA, Abrams K, Timmis A, et al. Prognosis research strategy (PROGRESS) 1: a framework for researching clinical outcomes. BMJ. 2013;346:e5595. 23386360 10.1136/bmj.e5595 PMC3565687

[15] Hingorani AD, Windt DA, Riley RD, Abrams K, Moons KG, Steyerberg EW, et al. Prognosis research strategy (PROGRESS) 4: stratified medicine research. BMJ. 2013;346:e5793. 23386361 10.1136/bmj.e5793 PMC3565686

[16] Riley RD, Hayden JA, Steyerberg EW, Moons KG, Abrams K, Kyzas PA, et al. Prognosis research strategy (PROGRESS) 2: prognostic factor research. PLoS Med. 2013;10(2):e1001380. 23393429 10.1371/journal.pmed.1001380 PMC3564757

[17] Steyerberg EW, Moons KG, van der Windt DA, Hayden JA, Perel P, Schroter S, et al. Prognosis research strategy (PROGRESS) 3: prognostic model research. PLoS Med. 2013;10(2):e1001381. 23393430 10.1371/journal.pmed.1001381 PMC3564751

[18] Sterne JA, White IR, Carlin JB, Spratt M, Royston P, Kenward MG, et al. Multiple imputation for missing data in epidemiological and clinical research: potential and pitfalls. BMJ. 2009;338:b2393. 19564179 10.1136/bmj.b2393 PMC2714692

[19] Harrell JFE. Regression modeling strategies: with applications to linear models, logistic and ordinal regression, and survival analysis. Springer series in statistics, 2nd ed. Cham: Springer International Publishing: Imprint: Springer; 2015:1 online resource (XXV, 582 pages 157 illustrations, 53 illustrations in color.

[20] Steyerberg EW, Vergouwe Y. Towards better clinical prediction models: seven steps for development and an ABCD for validation. Eur Heart J. 2014;35(29):1925–31. 24898551 10.1093/eurheartj/ehu207 PMC4155437

[21] Tran A, Lamb T, Taljaard M, Fernando SM, Inaba K, Moore EE, et al. Current practices and challenges in assessing traumatic hemorrhage: an international survey of trauma care providers. J Trauma Acute Care Surg. 2021;90(5):e95–100. 33891575 10.1097/TA.0000000000003081

[22] Savage SA, Sumislawski JJ, Zarzaur BL, Dutton WP, Croce MA, Fabian TC. The new metric to define large-volume hemorrhage: results of a prospective study of the critical administration threshold. J Trauma Acute Care Surg. 2015;78(2):224–9. 25757105 10.1097/TA.0000000000000502

[23] Savage SA, Zarzaur BL, Croce MA, Fabian TC. Redefining massive transfusion when every second counts. J Trauma Acute Care Surg. 2013;74(2):396–400. 23354230 10.1097/TA.0b013e31827a3639

[24] Ho AM, Dion PW, Yeung JH, Joynt GM, Lee A, Ng CS, et al. Simulation of survivorship bias in observational studies on plasma to red blood cell ratios in massive transfusion for trauma. Br J Surg. 2012;99(Suppl 1):132–9. 22441868 10.1002/bjs.7732

[25] Yang S, Mackenzie CF, Rock P, Lin C, Floccare D, Scalea T, et al. Comparison of massive and emergency transfusion prediction scoring systems after trauma with a new Bleeding Risk Index score applied in-flight. J Trauma Acute Care Surg. 2021;90(2):268–73. 33502145 10.1097/TA.0000000000003031

[26] Cowley LE, Farewell DM, Maguire S, Kemp AM. Methodological standards for the development and evaluation of clinical prediction rules: a review of the literature. Diagn Progn Res. 2019;3:16. 31463368 10.1186/s41512-019-0060-y PMC6704664

[27] Steyerberg EW, Mushkudiani N, Perel P, Butcher I, Lu J, McHugh GS, et al. Predicting outcome after traumatic brain injury: development and international validation of prognostic scores based on admission characteristics. PLoS Med. 2008;5(8):e165. 18684008 10.1371/journal.pmed.0050165 PMC2494563

[28] Trauma ACoSCo. ACS TQIP Best practices guidelines in imaging. 2018:51–3

[29] Ghossein J, Fernando SM, Rochwerg B, Inaba K, Lampron J, Tran A. A systematic review and meta-analysis of sample size methodology for traumatic hemorrhage trials. J Trauma Acute Care Surg. 2023;94(6):870–6. 36879398 10.1097/TA.0000000000003944

[30] Bleeker SE, Moll HA, Steyerberg EW, Donders AR, Derksen-Lubsen G, Grobbee DE, et al. External validation is necessary in prediction research: a clinical example. J Clin Epidemiol. 2003;56(9):826–32. 14505766 10.1016/s0895-4356(03)00207-5

