# Supplementary material for: The revised Canadian Bleeding (CAN-BLEED) score for risk stratification of bleeding trauma patients: a mixed retrospective—prospective cohort study
Source: Scand J Trauma Resusc Emerg Med. 2025 Feb 20;33:31. doi: 10.1186/s13049-025-01336-z (PMC11844109; doi:10.1186/s13049-025-01336-z)
Supplement: Supplementary file 1 [file 13049_2025_1336_MOESM1_ESM.docx]

**Supplement**

Summary of the Full Prediction Model with the Score Thresholds

| **Variable** | **OR** | **95% CI** | |
| --- | --- | --- | --- |
| *Initial SBP* – 110 vs 90 mmHg | 0.54 | 0.34 | 0.85 |
| *Initial HR –* 120 vs 100 bpm | 1.63 | 1.25 | 2.13 |
| *Lactate > 5 mmol/L* | 2.24 | 1.34 | 3.75 |
| *Penetrating Mechanism* | 1.85 | 1.21 | 2.83 |
| *Unstable Pelvis* | 3.10 | 1.64 | 5.88 |
| *FAST Positive for Free Fluid* | 3.64 | 2.38 | 5.56 |
| *External Bleeding* | 7.98 | 4.49 | 14.18 |
| *CT Positive for Free Fluid or Contrast Extravasation* | 4.32 | 2.97 | 6.28 |

**SBP: Systolic Blood Pressure; HR: Heart Rate; FAST: Focussed Abdominal Sonography for Trauma; CT: Computed Tomography**

**Flexible Modeling of Initial Systolic Blood Pressure using Restricted Cubic Splines**


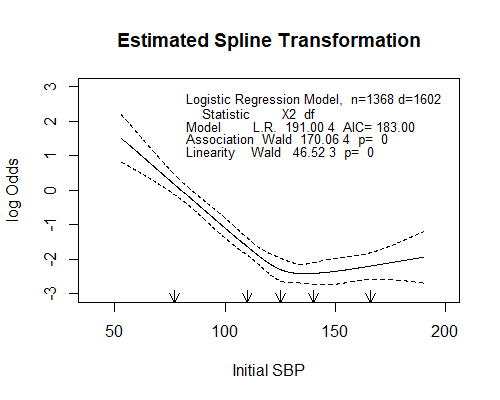


**Flexible Modeling of Initial Heart Rate using Restricted Cubic Splines**


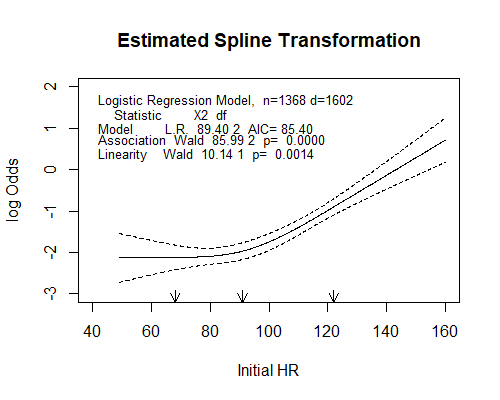


**Expected vs Observed Risk**

| **Score** | **Total** | | **Expected outcomes** | | **Observed outcomes** | |
| --- | --- | --- | --- | --- | --- | --- |
|  | **N** | **%** | **N** | **%** | **N** | **%** |
| **0** | 407 | 29.8% | 7 | 1.8% | - | 0.0% |
| **1** | 262 | 19.2% | 10 | 4.0% | 7 | 2.7% |
| **2** | 211 | 15.4% | 18 | 8.4% | 22 | 10.4% |
| **3** | 175 | 12.8% | 30 | 17.0% | 41 | 23.4% |
| **4** | 112 | 8.2% | 35 | 31.5% | 38 | 33.9% |
| **5** | 83 | 6.1% | 42 | 50.6% | 40 | 48.2% |
| **6** | 62 | 4.5% | 43 | 69.6% | 43 | 69.4% |
| **7** | 42 | 3.1% | 35 | 83.7% | 32 | 76.2% |
| **8** | 10 | 0.7% | 9 | 92.0% | 7 | 70.0% |
| **9** | 3 | 0.2% | 3 | 96.2% | 3 | 100.0% |
| **10** | 1 | 0.1% | 1 | 98.3% | 1 | 100.0% |
| **11** | - | 0.0% | - | 99.2% | - | -- |
| **12** | - | 0.0% | - | 99.7% | - | -- |
